# Assessing Credibility: Quality Criteria for Patients, Caregivers, and the Public in Online Health Information—A Qualitative Study

**DOI:** 10.1177/23743735241259440

**Published:** 2024-05-31

**Authors:** Lubna Daraz, Cicek Dogu, Virginie Houde, Sheila Bouseh, Knondoker G Morshed

**Affiliations:** 1 School of Library and Information Science, Faculty of Arts and Sciences, 5622 University of Montreal, Montreal, Québec, Canada; 2 Independent Researcher, Hamilton, Ontario, Canada; 3 Independent Researcher, Dhaka, Bangladesh

**Keywords:** Internet, health information, reliability, patients, quality criteria

## Abstract

The increasing reliance on the Internet for health information has raised concerns about patients using unreliable and potentially harmful content. This study aimed to establish quality criteria to assist patients, caregivers, and the public in evaluating the reliability of online health information. We conducted focus group workshops with 25 participants recruited across Canada, proficient in either English or French. The participants included 13 females and 12 males, with the majority having a college or higher level of education. Through an in-depth analysis comparing various aspects, the participants determined 6 quality criteria: authorship, reliability, usefulness, accessibility, readability, and privacy & confidentiality. The findings from this study present a comprehensive list of quality criteria that will contribute to developing evidence-based quality benchmarks and policy frameworks in multiple languages. These criteria are not only valid but also well-suited to the diverse needs and preferences of patients and the public, providing a reliable framework for evaluating online health information through an evidence-based approach.

## Key Points

- Patients and the public often face challenges in discerning between accurate and misinformation on the web due to a lack of knowledge and health literacy.
- Study participants overwhelmingly expressed a lack of awareness of the quality standards essential for distinguishing between reliable and unreliable health information online.
- The study engaged diverse patients, caregivers, and the public in collaboratively developing a unique set of quality criteria.
- The quality criteria aim to empower individuals by equipping them with the skills needed to actively engage in and navigate their healthcare more effectively.
- The quality criteria may also serve as a guideline for clinicians and online health information providers, ensuring that individuals can access trustworthy health information on the Internet.

## Introduction

With the explosion of health information readily available online, the question of validating this kind of information has become paramount. It is a fact that trustworthy health information (HI) has the potential to change behavior, enhance adherence to treatment, minimize risks, increase satisfaction with care, and ultimately enhance health outcomes.^1-4^ Despite the presence of numerous online sources, there are still concerns regarding its reliability. It is quite possible that inaccurate HI can cause harm to patients and the public, and could potentially have an adverse impact on healthcare systems. Some harms include poor health outcomes, misleading interpretations, increased return visits, and tremendous resource waste.^5-7^

On Google alone, about 7 million searches are performed daily for health-related information globally.^8^ The COVID-19 pandemic has thus led to an unprecedented “infodemic,” making it crucial to ensure that online HI (OHI) is based on evidence.^1,2,9-14^ This infodemic has also contributed to the proliferation of misinformation and the subsequent consumption by Internet users seeking OHI. According to a study conducted in the United States, 68.9% of participants used the Internet to access HI.^4^ It has also been reported that 80% of the patients, caregivers, and the public (PCP) research their doctors’ recommendations on the Internet after consultation to better understand these recommendations.^15^

Nevertheless, the potential for web-based information to positively influence health and health equity is undeniable. The potential benefit is contingent on PCP and those involved in facilitating health, who can find relevant information and discriminate between high quality and misinformation. Extensive access to reliable HI provides the opportunity to inform, teach, and connect healthcare professionals and patients. The widespread use of the Internet as a source of HI has thus become a double-edged sword for PCP and clinicians. On the one hand, it provides easy access to a wealth of information, but on the other hand, it also means that they are at risk of having access to harmful information.^5,6,16-19^

It is therefore difficult for PCP to know what to believe. If they act on wrong information, their health may worsen, including the loss of lives.^10,11,16^ A rapid review that investigated the current state of the OHI relevant to COVID-19 demonstrated that misinformation had a negative impact on health outcomes and human lives.^16^ Another study conducted by the Reuters Institute in 2020, which surveyed 6 countries (Argentina, Germany, South Korea, Spain, the United Kingdom, and the United States), showed that one-third of online users encountered false or misleading information about COVID-19.^13^ The authors highlighted a concerning trend: individuals with low health literacy levels are more inclined to depend on the Internet for information compared to other sources. As a consequence, as more PCP turn to the web to address their HI needs, there is a higher risk of encountering unreliable information from deceptive HI producers. In addition to the poor readability (a major criterion for quality), many websites have nonevidence-based and biased information due to the writers’ or their sponsors’ financial and intellectual conflicts of interest.^5,8,19^ As a result, there is an urgency to address this issue with the likelihood of future pandemics, climate change, and other public health emergencies.

To tackle this pressing issue, we have embarked on an ambitious research endeavor aimed at developing evidence-based tools and policy guidelines.^20^ The research steps include: (1) 2 systematic reviews to evaluate the current state of the quality of OHI and identify existing OHI assessment tools^5,21^; (2) content analysis of selected OHI assessment tools and draft a set of initial quality criteria^22^; (3) conduct focus groups to determine and validate a final list of quality criteria; (4) develop and validate quality benchmarks and policy frameworks in multiple languages with a large sample; and (5) disseminate and implement.

The findings of the systematic reviews (step 1) demonstrated that the majority of the websites that provide HI for PCP, are of low quality and they require college or higher level of education to read, understand, and apply OHI.^5,21^ Based on these findings, we conducted a content analysis (step 2) of 17 selected HI assessment tools (i.e., benchmark, checklist, codes of conduct, etc.) identified from the systematic reviews.^22^ Our analysis revealed many limitations with these tools. For example, most of the tools are not easily accessible by PCP, are poorly designed, are not patient-centered, have questionable quality, and require a high level of literacy to use. Moreover, our synthesis failed to identify a gold standard that was developed and validated through active participation by diverse PCP.^23^ The tools comprise quality principles, such as “reliability,” “credential,” or “usefulness” that are used to assess the level of trustworthiness. These principles are accompanied by corresponding questions that represent the underlying theme. We refer to these principles as “Quality Criteria” and the accompanying questions as “Questions” that describe each quality criterion.

During the content analysis of the selected tools, the team formulated 92 quality criteria and devised 223 questions for additional assessment.^22-25^ The criteria were then condensed into fewer categories by identifying both overlapping and nonoverlapping themes. It is worth noting that many of the existing evaluation tools were developed for specific user groups and often lacked input from a diverse range of individuals (i.e., PCP).^22^ In this article, we describe step 3 of our multifaceted project that involved conducting a qualitative study with active participation by PCPs to determine a list of unique quality criteria, offering a guideline to help them assess the reliability of OHI. This phase represents a pivotal milestone within our broader research program.

## Method

### Study Design

Workshops utilizing focus groups with a diverse range of PCPs were employed to gather qualitative data.^26^ This approach enables the acquisition of detailed and nuanced information through active engagement with participants, proving to be both efficient and cost-effective—attributes frequently overlooked in survey-based or other data-driven research methods.^26-28^ Utilizing a focus group approach in a workshop format may also lead to interactions between individuals that provide additional insight and a deeper understanding of the phenomena being studied. In addition, a list of semistructured interviews was carried out with a varied group of policy and decision-makers, encompassing clinicians. The outcomes of these interviews will be presented in an upcoming publication.

### Study Participants

We employed purposeful criterion sampling to select participants who could offer the most insightful information about the phenomenon being studied.^26,28^ For the purpose of the study, it was crucial to choose individuals with Internet access and prior experience in searching for OHI. To ensure that they could provide relevant and substantial insights into their preferences for quality criteria, we established the following inclusion criteria: (1) women and men, (2) aged 18 years or older, (3) have either personally dealt with an illness or served as a caregiver, (4) have access to the Internet, (5) have experience searching for OHI 1 year or more, and (6) can communicate in English or French. We excluded participants based on the following criteria: (1) do not have experience with OHI seeking, (2) do not have access to computers and the Internet, and (3) healthcare professionals or experts. We made an effort to include participants from a wide range of socioeconomic backgrounds, encompassing variables such as gender, race, age, social class, income, education, and employment status.

### Recruitment Strategies

The study was conducted in the French Province of Québec in Canada. Therefore, we recruited participants in both official languages (English and French) abilities. Participants were identified and recruited through gatekeepers for PCPs in Québec Province and the rest of Canada. These gatekeepers included various organizations such as patients’ engagement units at affiliated hospitals of the University of Montreal, patients’ advocacy groups, public health education centers, community centers for immigrants, support groups, and local public libraries in Quebec and across Canada. The study coordinator contacted these organizations to introduce them to the study and extend invitations to participate. Upon agreement to participate, the organizations were provided with a study recruitment flyer approved by the Ethics Committee. This flyer was used to advertise the study within these organizations and to recruit participants.

We also used a snowball sampling approach to ensure equitable representation of participants with characteristics that may be underrepresented in the focus groups.^26^ The study was approved by the University of Montreal's Research Ethics Board, the Comité d’éthique de la recherche en arts et humanités (CERAH) and all participants provided written informed consent prior to study enrollment. An honorarium was provided to each participant as a token of appreciation for their participation.

### Data Collection

Two focus groups were conducted in Montreal, Canada. The method used for focus groups was web conference workshops as the study was conducted during the coronavirus pandemic. The duration of the workshops was 4 h with frequent breaks. Three research personnel with both language abilities conducted the workshops.

During the workshop, the study participants engaged in interactive discussions after being given a concise educational presentation. The presenter used a PowerPoint guide that contained a list of quality criteria and examples from healthcare websites. The workshops covered the following topics: (1) an overview of OHI, (2) the advantages and disadvantages of OHI, (3) the current state of OHI, (4) a brief introduction to quality assessment tools, and (5) a detailed explanation of each quality criterion along with its corresponding definition, which was selected based on the earlier content analysis (step 2) and established sources.^21,22,29-35^

Prior to the focus groups, a questionnaire was distributed to collect demographic data. Throughout the study, the confidentiality of the participants was preserved. Regarding ethnicity, we asked open-ended questions, and participants self-identified their identity. Therefore, we classified ethnicity based on the participant's own description. To ensure participants’ confidentiality, no personal or identifiable information was requested, and participants were given the option to turn off their cameras during the workshops.

We presented the participants with an extensive list of quality criteria and corresponding questions identified from the prior content analysis of the selected tools.^22^ After an interactive discussion about the criteria, participants were asked to express their preferences and vote on a list of criteria they deemed most important in evaluating the quality of OHI. They were also asked to identify any other criteria they believed were crucial for assessing the quality of OHI, which we might have missed. However, they did not bring up any additional criteria; instead, they affirmed that the list we presented to them was comprehensive.

Using the voting results, we selected the most popular quality criteria and questions for each language group. Additionally, participants provided feedback on the format, readability, and scoring criteria, which will be discussed in a forthcoming publication. At this point, we determined that data saturation was reached from the rich, interactive discussions among the participants that led to the identification of the final list of the quality criteria.^26,28^

### Data Analysis

During the workshops, we conducted the analysis simultaneously with the collection of discussion data. The constant comparison analysis, which is commonly used for multiple focus groups within the same study, was employed.^36^ This analysis consisted of 5 stages: firstly, we transcribed the workshop audio recordings verbatim. Then, 2 study personnel independently read and re-read the transcripts. Next, we compared the participants’ choice of quality criteria with their vote and the phrases spoken by study participants to understand the ‘why’ and ‘how’, presented below. We reviewed and selected participants’ direct quotes that corresponded with their choice of quality criterion. Finally, we drafted a list of quality criteria and a choice of questions based on the highest votes received for each quality criterion. We used NVivo 12 Pro for the data analysis.

## Findings

### Demographics

The study involved 25 participants aligning with the suggested range for an ideal focus group size, which typically falls between 8 and 10 participants.^28^ The composition reflected a balanced distribution in terms of gender, with 13 females and 12 males. The age range of the participants varied from 18 to 44 years. Notably, a majority of the participants held a college or higher level of education. All participants had more than 5 years of experience in searching for OHI. The representative sample contributed to a comprehensive exploration of the topic under investigation, providing valuable insights and perspectives about the final list of the quality criteria. The demographic characteristics of the participants are presented in Table 1.

**Table 1.** Focus Group Demographics (N = 25).

Demographics	Total participants (n** **=** **25)
***Gender***	
Female	13
Male	12
***Age***	
18-24	9
25-34	10
35-44	6
***Language spoken***	
English	14
French	11
***Education***	
Secondary	7
Higher	18
***Employment status***	
Full-time	7
Part-time	4
Unemployed	14
***Income***	
$25K or less	4
$26K-50K	8
$51K-85K	6
$86K-125K	6
$125K+	1
***Ethnicity***	
Caucasian	9
Latino	2
Asian	8
Mixed	6

### Selected Quality Criteria

The definitions for each quality criterion used in the workshops were taken from established sources where available. Below, we describe the criteria set along with feedback from participants to demonstrate their perceived importance.

### Authorship

To ensure *Authorship*, the names of the authors, contributors, and credentials should be mentioned in the information resource.^19^

*It's important to have credentials … I think when it comes to health, if you read something that's especially about cancer, if it comes from, like, a doctor, I think it will give you more credibility.*

However, participants also highlighted further important aspects to consider, for example, the author's potential biases:

*… they'll write stuff, but it will be very biased … So it won't be like a neutral opinion or like a neutral article or information on how to help people. While someone can have the credentials, I've also seen it before that they can switch it around and it won't be as accurate and beneficial to everyone.*

*I think this question is really relevant and quite clear, and I think we do need qualified professionals, especially when they're giving advice regarding cancer and all.*

### Reliability

Despite the absence of an established definition in the literature,^35^ *reliability* is deemed to be “the consistency and dependability of the information.”^37^ Participants confirmed that reliability was crucial to evaluating the quality of OHI:

*I think it is really important to have concise information and also talking about the diagnosis and also the treatment and the health risk factors.*

*Having information up to date is especially important, especially in the age of COVID, because what we know about COVID has been constantly changing.*

*… someone who doesn't know much about medicine and the scientific evidence and all that, it would be pretty hard for me to know if there is really scientific evidence.*

*I think this is quite a relevant question because in theory, I would expect all of the medical information that I'm presented to be neutral and unbiased, so I can just trust on it.*

### Usefulness

*The usefulness* of the content of OHI is related to its appropriateness with the target audience and the fact that it supports PCP's need. According to the participants, they should be able to understand this kind of information easily and without any technical knowledge:

*It's difficult to use the information if you do not understand the medical terms they are using. If one can’t understand the information, it may lead to wrong self-diagnosis.*

*I think that we must first determine what the target audience is and then, to see if the information matches, what type of people would be looking for there.*

*I feel like this question is quite relevant in terms of usefulness, mainly because if the information is not appropriate for the target audience, then I don't think, like, what is the point of the article if it's not even catered to the target audience?*

### Accessibility

The importance of easy access to available and usable (*accessibility*) OHI was also highlighted as very essential:

*I wouldn't like to have a hidden link. I prefer everything to be accessible through the menus that are available on the pages of the website.*

*… it's important to have some sort of contact information, but I think having the phone number, email provided is too much, especially with all these things now … Maybe just the email address enough*.

*I think it's very relevant important to address those accessibility needs and make sure the information can be accessed.*

### Readability

*Readability* is a “measure of the ease with which a passage of text can be read.”^21,30^ The participants emphasized that the information should be presented clearly and understandably, catering to the level of comprehension of lay PCP:

*I think it also depends on the target audience, like, writing to the general public. We must use public language.*

*I think a simpler way could just be can you understand the information?*

### Privacy & Confidentiality

As per the participants, a website providing HI must uphold the *privacy & confidentiality* of its visitors. Additionally, it should offer the option of anonymous navigation, without requiring users to disclose their personal information on the site:

*… yes, we ask this question, but on what we base ourselves to be able to conclude that the company respects privacy? It is often a confidentiality code or a confidentiality policy. First is it accessible on the website? Then is it understandable by the patients?*

*The only thing I'll be concerned is to share too much information about myself, especially my medical card and address. So I don't think why they have to grab all this information.*

The final list of the quality criteria can be found in Table 2, along with the corresponding questions selected by the study participants.

**Table 2.** Quality Criteria and Questions Selected by the Focus Group Participants.

Quality Criteria	Questions
Authorship	Can you tell if qualified professionals are giving the advice? (For example: Physicians, Nurses etc.)
Reliability	Is the date of the information up-to-date? Are the benefits and harms associated with the information (health risk factors, diagnosis, treatment, etc.) presented? Can you tell if the information is written based on nonbiased scientific evidence?
Usefulness	Do you find the information usable? Is the information appropriate for target audience?
Accessibility	Can you tell whether the information is for commercial or educational purpose? Can you find the information by following links from the home page? Does it provide details of additional sources of support?
Readability	Is the information easily understandable for your use?
Privacy & Confidentiality	Does the website respect privacy & confidentiality of visitors? Can you use the website anonymously?

No significant variations were observed among participants in different languages in terms of the quality criteria. However, there were some disparities between language groups in relation to the accompanying questions. For example, regarding the criterion ‘Authorship,’ French-speaking participants included 2 additional questions: (1) Is it possible to trace the author of the information? and (2) Are the name and contact information of the organization available on the web pages? Therefore, we anticipate developing the quality criteria in multiple languages.

## Discussion

As the usage of online platforms continues to rise, there is a growing need for new educational models that enable patients and their caregivers as well as members of the public to share medical knowledge. It is crucial to equip individuals with effective strategies for navigating and identifying misinformation on the web. Our objective in this qualitative study was to identify and validate a unique list of quality criteria. The purpose of these criteria is to develop evidence-based quality benchmarks and policy frameworks for OHI providers in multiple languages, which can aid diverse PCP in evaluating the trustworthiness of OHI.

Each of the selected quality criteria was discussed in-depth and recommended by the participants. For example, *authorship* is a major concern that has also been noted in previous research.^6,31^ The relationship between the quality of OHI and the disclosure of authorship is essential for the dissemination of medical information on the Internet. A study conducted by Liu et al^32^ emphasized that individuals are responsive to potential biases of authors and are less inclined to utilize OHI that is produced by a prejudiced author. This partiality was seen as linked to the author's influence. Our study therefore confirmed that a health site can be considered credible if the credibility of the author is clearly stated. Liu et al^32^ also stated that the owner of the source of the information was “one of the most widely reported indicators consumers applied to evaluate the quality of online health information”.

Another crucial aspect in assessing the quality of OHI, often used interchangeably with credibility, is *reliability*. This was considered a leading aspect by the participants, given the dynamic nature of medicine. They expressed the fear that they often lacked the necessary skills and medical knowledge to evaluate the reliability of OHI. This observation is in line with the earlier findings where the authors reported that most of the websites do not meet the minimum standard for quality of OHI targeted to PCP.^5^ *Usefulness* is another criterion closely linked to quality. The study participants believed that they should be able to comprehend the type of information without any technical expertise. They also noted that individuals may find it challenging to assess and use evidence-based content on the web.

*Accessibility*, a major component of quality was also identified and preferred as crucial by the study participants.^33^ The participants in this study emphasized the importance of ensuring OHI is easy to access. This aligns with previous research, which also highlighted the significance of *readability* matching the target audience and presenting HI in a way that individuals can easily understand and apply.^21,30,34^ It is worth noting that our quality criteria are the first guidelines to include the important criterion of *readability*, a factor overlooked by previous tools. Finally, this study demonstrated the importance of *privacy and confidentiality* when PCPs navigate health websites. The participants expressed their concerns regarding the need for anonymity during website navigation and emphasized that a website's confidentiality policy should be readily available and accessible to its users.

### Limitations

The majority of the participants had a higher level of education, and individuals aged 45 and above were not adequately represented in the sample. This limitation will be addressed in the next stage (step 4) of our research which involves conducting surveys in Canada and the United States with a larger sample to ensure the generalizability of the intervention. This approach aims to ensure that the intervention accounts for the diverse needs and preferences of various user groups before it is disseminated and implemented to a broader population.

## Future Direction

This qualitative study constituted a significant component of a larger research program focused on examining the quality of OHI and developing evidence-based solutions to assist diverse PCPs in distinguishing reliable information from unreliable sources.^20^ Building upon the findings presented in this article, currently we are conducting “Member Checking” and “Usability Test” with a representative sample to further validate the quality criteria. The outcomes of these evaluations, alongside any necessary adjustments to the quality criteria and accompanying questions, will be employed to conduct international surveys with a larger sample.

## Conclusion

As the use of online sources continues to grow, there is a rising need for novel learning strategies that enable diverse patients and members of the public to access and utilize reliable health information. To date, no one has developed evidence-based and user-friendly solutions with and by diverse stakeholders. The variation in the reliability of the existing tools thus justifies the need for a universal, validated, user-centered tool to address the spread of health misinformation on digital platforms. This study has developed a set of quality criteria based on the active stakeholders’ participation, aimed at assisting them in accessing credible health information from the web, ultimately leading to improved health outcomes.
